# ‘What does “remaining living at home” really mean?’ Stakeholders’ perspectives on everyday difficulties among older people with chronic conditions

**DOI:** 10.1177/03080226241306326

**Published:** 2024-12-20

**Authors:** Thomas Ballmer, Brigitte Elisabeth Gantschnig

**Affiliations:** 1 Institute of Occupational Therapy, School of Health Sciences, ZHAW Zurich University of Applied Sciences, Winterthur, Switzerland; 2 Department of Rheumatology and Immunology, University Hospital (Inselspital), University of Bern, Bern, Switzerland

**Keywords:** autonomy, chronic illness, occupational therapy, older people

## Abstract

**Introduction:**

Older people with chronic conditions prioritize living arrangements that let them maintain their autonomy. However, many face activity limitations and participation restrictions. Stakeholders involved in their health and social care have a unique perspective on these challenges. The aim of this study was to examine how these stakeholders view the difficulties older people with chronic conditions living at home face in their everyday lives.

**Method:**

We conducted a qualitative descriptive study based on a focus group interview with eight stakeholders involved in health care, social care and housing for older people with chronic conditions. We transcribed the interview verbatim and analysed it using thematic analysis.

**Findings:**

We generated four themes: fear of losing autonomy, inequality along socio-economic lines, persisting and new barriers, and ‘what does “remaining living at home” really mean?’. Participants problematized the lack of awareness among key stakeholders for older people’s needs and limited housing options beyond the dichotomous choice between ageing at home and in a care home.

**Conclusion:**

Acknowledging diverse factors – fear of losing autonomy, socio-economic situation, limited awareness of key stakeholders, physical and other barriers, societal discourses – shaping older peoples’ choices regarding their living arrangements informs how occupational therapists can support them.

## Introduction

Considering current demographic trends, the number of people aged 65 years or older in the European Union will reach 149 million in 2050 (Eurostat, 2019). The number of people aged 85 years or older is projected to grow even more rapidly, from 13.8 million in 2018 to 31.8 million in 2050 (Eurostat, 2019). Similarly, in the United Kingdom (UK), the number of people aged 80 or over is projected to grow by 108.7% between 2023 and 2063 (Centre for Ageing Better, 2023). The vast majority of these people are expected to be living in private households. The most recent European census data show that as of 2011, 96.4% of persons 65 years or older and 86.7% of persons 85 years or older lived in private homes (Eurostat, 2014). The majority of older people living in private homes live with chronic conditions associated with limitations in their activities of daily living, as well as restrictions in their participation at home and in the community (Bähler et al., 2015; Griffith et al., 2016; Henchoz et al., 2017).

In the Madrid International Plan of Action agreed upon at the Second World Assembly on Ageing in April 2002, the United Nations have voiced their support for policies promoting ageing-in-place (United Nations, 2002), including improving the accessibility of housing and public spaces and improving older peoples’ access to goods and services. In Switzerland, the support of ageing-in-place for older people is also codified in several policy frameworks on a national, cantonal and communal levels (Bundesrat, 2007; Dienststelle Soziales und Gesellschaft Luzern, 2010; Gemeinde Kriens, 2015). In order to design interventions that support older adults with chronic conditions to continue living in the surroundings of their choice, we need to know more about the actual challenges and difficulties they encounter in everyday life.

### Literature review

In the literature, we do find a number of studies on older people’s own views on everyday difficulties. A recent study examined how older people interpret, navigate and overcome such difficulties (Ballmer and Gantschnig, 2023). For the older people in that study, maintaining their autonomy and agency in the face of everyday difficulties was more important than to merely remain living in the house or apartment that they are used to. Autonomy in this instance is not the same as independence, but rather ‘the ability and opportunity to act in accordance with one’s values, unfettered by coercion, duress, restraint or deceit’ (Norman, 2005). The study also found that older people were using highly individual, creative strategies to navigate everyday difficulties, including accessibility issues in their homes and public spaces, access to formal and informal support, and inadequate funding opportunities for adaptations (Ballmer and Gantschnig, 2023). This importance of maintaining autonomy is supported by the findings of other studies in this field (Eloranta et al., 2008; Haak et al., 2007).

Ewart and Luck (2013) conducted an ethnographic study with British older adults who described the importance of participation outside to their participants, and the barriers making this more difficult for them (Ewart and Luck, 2013). However, facilitators and barriers to autonomy for older people have been described more extensively for people living in residential care facilities, mainly in terms of characteristics of the institutions in question (van Loon et al., 2021; Welford et al., 2012). Relatively little is known about what affects the autonomy of older people with chronic conditions living at home. How other stakeholders in the provision of health care, social care and appropriate housing to older people with chronic conditions view the everyday difficulties that this group faces could complement older peoples’ own subjective views and contribute to a more complete picture of their situation. This could inform the design of services for older people that are appropriately embedded in local political and institutional structures. In Switzerland, this is all the more important, due to the high degree of fragmentation within the health care system and the uneven accessibility of auxiliary support services for older people, grounded in Swiss political federalism (Bundesrat, 2019; Höpflinger et al., 2019).

In order to complement older peoples’ subjective understanding of the everyday difficulties they face and how to navigate them, our study, therefore, aims to examine how different stakeholders involved in health care, social care and housing for older people with chronic conditions view the difficulties, these people face in their everyday lives.

## Method

### Theoretical background

In this qualitative descriptive study (Sandelowski, 2000, 2010), while still situated in an interpretive paradigm (Denzin and Lincoln, 2008), we pursue a pragmatic approach to the generation of knowledge, as the goal was to identify current problems and resources that could help or hinder the development of services for older people with chronic conditions living at home.

### Study design and setting

The community where we carried out the study is situated in a suburban area close to a medium-sized Swiss city. We purposively sampled participants to represent a range of relevant sectors (e.g. health care, social care and housing). In order to facilitate participants taking a broader view on the topic of discussion, we chose focus group methodology (Krueger and Casey, 2014).

We organized this focus group in tandem with a second focus group of older people with chronic conditions living at home (Ballmer and Gantschnig, 2023). The focus groups took place in a space provided by our project partners, a home nursing agency, in June of 2020. Both the older people in that study and the stakeholders chosen for this study live and/or work in the same community. The first and last author, who both had experience in conducting focus groups, planned and carried out both groups. We submitted the study protocol to the *Ethics Committee for Northwestern and Central Switzerland (EKNZ)* and were registered under the *Project-ID Req-2019-01159*. The EKNZ deemed it not in need of formal ethical approval as it was by its content not subject to the Swiss Human Research Act .However, according to the EKNZ, the study protocol nevertheless complied with general principles of ethical research involving humans.

### Participant recruitment

To prepare the recruitment of local participants from a range of different relevant sectors, the project team held brainstorming sessions to come up with eligible organizations and persons from the health care, social care and housing sectors, as well as seniors’ organizations and local/regional government. The first author conducted additional online research. Different members of the project team then contacted 15 organizations and/ or persons directly via e-mail. Nine persons agreed to take part in the focus group. Of these nine, one person did not attend the focus group for unknown reasons. The inclusion requirement for participants was that they worked in a field relevant to older people with chronic conditions living at home in the region. No exclusion criteria were defined.

### Data collection

Prior to the focus group, we provided participants with written information on the aim of the study, what participating in the focus group would entail, how the data would be used and their right to withdraw from the study at any point via e-mail. On the day of the focus group, they gave written consent and were asked to fill out a short form to collect basic sociodemographic information. The focus group interview took place in a meeting room that was centrally located in the community and lasted for approximately 120 minutes. An interview guide was developed to facilitate discussion on how different stakeholders understand the difficulties older people with chronic conditions living at home face in their everyday lives. We formulated the following general questions:

- In your view, what are the greatest challenges for older people with chronic conditions living at home and their relatives (a) to remain living at home for as long as they would like and (b) to be able to keep up their meaningful occupations (i.e. activities that are important to them)?
- Based on your experience, which forms of support can best help these persons (a) to remain living at home for as long as they would like and (b) to be able to keep up their meaningful occupations doing so?
- What do you see as the potential of and the difficulties with small- and large-scale home adaptations in this process?

We digitally recorded the focus group interview and transcribed it verbatim. The first author double-checked the transcripts and uploaded the audio files and transcripts to a secure server.

### Data analysis

In keeping with our pragmatic approach, we decided to use thematic analysis (Braun and Clarke, 2012) to analyse the data. After listening to the recording and rereading the transcript repeatedly, the first author inductively coded units of meaning within the text relevant to the research question. The last author also coded a portion of the transcript to increase credibility. The first author then reviewed the codes and identified four common themes. In keeping with our pragmatic approach, generation of codes and themes was predominantly semantic. Furthermore, we examined the consistency of the themes with the data as a whole, and the first and last author discussed and adapted the themes to increase credibility. The first author then named, defined and described the themes, anchoring them in verbatim quotes from the transcripts.

### Trustworthiness

To bolster the trustworthiness, that is, the methodological rigour, of our research (Lincoln and Guba, 1985), we applied several methods, including researcher triangulation (as described above) throughout the analysis process, peer debriefing with members of our research group not directly involved in the project, documentation of our code and theme generation and adaption (Nowell et al., 2017). At the outset of the research project, the authors reflected on their positionality as white, able-bodied, middle European academics with regards to the topic of autonomous ageing and the diverse group of people it affects (Chaouni et al., 2021). All data analysis meetings between the authors included a reflexive appraisal of how their subjectivity and context may influence the process of code and theme generation (Phelan, 2011).

### Findings

Eight participants (six women and two men) took part in the focus group. They were between 39 and 75 years of age (mean = 45.7, SD = 13.1) and had an average professional experience in their respective field of 14.7 years (SD = 8.6). We provide more detailed information about the participants in Table 1.

**Table 1.** Participant characteristics.

Participant	Age in years	Gender	Profession	Area of work	Professional experience in current field in years
Anna	39	f	Social worker	Home-based counselling for older people (church-funded)	8
Barbara	46	f	Architect	Specialized in housing for older people	20
Clara	49	f	Home care nurse	Domestic home-based care for older people	5
Diana	55	f	Physical therapist/project manager	Project manager at non-profit health promotion organization	30
Eric	44	m	Social worker	Counselling for older people (church-funded)	16
Florian	58	m	Accessibility consultant	Accessibility counselling and advocacy for individuals and institutions	20
Gloria	32	f	Occupational therapist	In- and outpatient therapy with persons with neurological conditions	10
Hilda	75	f	Retired	Representative of local senior’s councils	–
Average (SD)	45.7 (13.1)	–	–	–	14.7 (8.6)

Our thematic analysis of the data yielded four themes: *the fear of losing autonomy, inequality along socio-economic lines, persisting and new barriers* and *‘what does “remaining living at home’ really mean?’*.

### Theme 1: The fear of losing autonomy

Several participants reported that they perceived the fear of losing autonomy as a common concern among old people. This fear seemed to not only inhibit people from taking advantage of health care services but also to inhibit them from taking on or even considering adaptations. Older people sometimes saw this as a first step in the process of eventually having to move to a home – as illustrated in the following quote:

We had many people who told their occupational or physical therapist they had stumbled or had suffered a fall: ’But don’t tell my doctor!’ It is always connected to a fear that it might lead to being committed to a nursing home. (Diana, physical therapist)

Admitting and addressing such problems in everyday life appeared to be an especially sensitive topic when relatives were involved, whereas professional advice was sometimes received more favourably. In the participants’ experience, many older people would put off adapting their home as long as possible, sometimes developing ‘alternative solutions’ in the process:

We had someone who used to slide up and down [the stairs] on the seat of their pants, because they were too afraid to walk. (Florian, accessibility consultant)

The participants saw the fear of losing autonomy as connected to the fear of falling and to general inactivity. Fear of falling could lead to avoidance behaviours and therefore to inactivity, whereas inactivity was a factor that made falls more likely. Inactivity was also seen as a factor for social isolation. In this way, behaviour and environment seem to be closely interrelated. The participants also named other possible reasons that could lead older people to not take advantage of services, assistive devices, or adaptions. A lack of imagination how these services, devices, or adaptations could help them was one of those reasons, as were feelings of inhibition to ask for help or ‘be a burden’ to others. The latter attitude was most attributed to the oldest generation (80 and over), while the “baby boomer” generation was seen as more used to advocating for their own needs.

However, for some people, the fear of losing autonomy – or the wish to maintain it – could also be a driving factor to seek support. For instance, to the participants, there seemed to be less and less reluctance to take advantage of home care services among older people. The participants connected this to a certain normalization of this kind of support in the public consciousness. The same applies to assistive devices. Instead of needing to contact a specialized organization or store, older people could now buy many of these devices at the supermarket, as Gloria, an occupational therapist, pointed out. This development goes along with a reduced stigmatization of such assistive devices and services. Some participants had experience with letting older people try out adaptations and assistive devices, themselves. This too was sometimes successful in lowering people’s inhibitions to use such devices.

When installed, adaptations often turned out to be not a threat to autonomy, but, on the contrary, something that enabled older people to regain autonomy. Even small adaptations (e.g., the installing of a handrail or better lighting) had the potential to decrease fear of falling and increase the general activity level of older people. For example:

One example is a woman who hadn’t left her house in two years [. . .] when someone finally noticed, and eventually a handrail was installed, she was able to leave the house again. But she wouldn’t say anything [. . .] (Clara, home care nurse)

Suddenly, things that seemed impossible before become possible again [. . .] When you screw in a brighter lightbulb and they suddenly see a lot more, small things like that [. . .] It certainly doesn’t work every time, but when it works it’s astounding, how little it takes sometimes. (Diana, physical therapist)

In summary, the fear of losing autonomy seems to be an important factor in how older people with chronic conditions decide to accept or refuse support. While this fear is often an inhibiting factor, especially if accepting adaptations or services is seen as a ‘first step towards the old people’s home’, it can sometimes also motivate people to be more open towards support. Participants perceived a certain normalization of adaptations and support services of older people in recent years.

### Theme 2: Inequality along socio-economic lines

Participants pointed out that inequality along socio-economic lines and the differences in resources available to older people are a decisive factor that shapes their access to adaptations and support services. Older people’s access to resources, especially financial resources, was seen as a major force influencing if and how older people with chronic conditions faced problems in their everyday life. Thus, their socio-economic situation determines if they will be able to adapt their homes to their needs. This is different from the situation in some other countries. Eric, a social worker, who had worked in the same field in Germany, pointed out that the barriers to home adaptation were markedly higher in Switzerland, due to the lack of funding and higher administrative hurdles:

The Pflegekasse [German care insurance] always paid a certain amount towards it [. . . .] the level of inhibition was much lower there, than here [in Switzerland], because of the cost, and all of the permits. . . (Eric, social worker)

This problem was not only seen in connection with adaptations or assistive technology. Some services, like domestic home care, are generally only reimbursed by health and social insurance for either those with private insurance, or those on government benefits.

Gloria also remarked that therapists struggle with the limited health insurance funding for accompanying adaptation processes. She described structural processes that can put pressure on people with less financial means to move to a nursing home instead of staying in their own home:

And with others, who had already faced more scarcity in their lives [. . .] and maybe also don’t have someone advocating for them at that moment, I often have the experience lately that there is an attempt to persuade them to move to a nursing home. Simply because there you have social contacts, there’s no thresholds, it’s barrier-free living. There, there is more of a tendency to steer people towards a nursing home. Because then, funding is available in different ways. (Gloria, occupational therapist)

Generally, participants agreed that it should be in the interest of health and social insurances and local, cantonal and national authorities alike to provide better funding in these areas. Participants felt that ethically, access to services should be available to people not based on if they have private insurance or government benefits, but based on what services they actually need. But even apart from such ethical concerns, participants were of the opinion that funding adaptations, assistive devices and access to services had the potential to prevent societal costs:

Finally, I think that the insurances should have an interest in this, because [. . .] when we had people with a thighbone fracture [. . .] those are ballooning costs that could be maybe avoided beforehand. (Clara, home care nurse)

In this theme, participants described how older people’s socio-economic position could determine the access they have to adaptations and services that would help them navigate challenges in everyday life and support their autonomy. Older people with less access to resources may face stronger structural pressure to move to an institution, regardless of their own wishes.

### Theme 3: Persisting and new barriers

Another recurring theme in the participants’ statements was that although there have been some developments in making public and private spaces more accessible, there were not only persisting barriers, but even new barriers that were arising, for instance connected to societal digitization.

Physical and other barriers to older people’s autonomy persist in public spaces and private homes alike. As examples, participants pointed to steps leading up to house entrances and inaccessible bathrooms. While older people’s own fears, attitudes and access to resources could prevent them from having adaptations made to their homes (as pointed out in themes 1 and 2), another factor could be some landlords’ reluctance to allow such alterations. For example, one participant remarked:

Installing handrails is something that people just don’t want to do, although it would benefit everybody. (Florian, accessibility consultant)

Also, in the participants’ experience, modern household appliances were often designed in a way which made them less rather than more usable for older people with chronic conditions. Gloria relayed one example referring usability of kitchen appliances for older people:

Try and find a new apartment where the kitchen appliances have no touch screens [. . .] I was at a kitchen appliances store [. . .] and they told me ‘the ones with the buttons, they won’t be produced anymore anyway’ [. . .] and then I said – I didn’t want to talk about old age, but blind people, how will they cook? ‘Oh, you can feel it here, to turn it on and off’. Well, turning on and off is not all you need to cook. (Gloria, occupational therapist).

In both areas, physical accessibility and product usability, participants see the need for raising awareness among government officials, landlords and manufacturers. This was not seen as merely an issue affecting older people. Participants stressed how accessible design could benefit everybody:

It’s the design for all that benefits all of us – from baby carriages to old age. (Florian, accessibility consultant)

Digitization is another development that, in the eyes of the participants, could create barriers for older people using services and products. This was seen in multiple areas. For instance, with more and more content moving online, older people who are less tech-savvy have more problems accessing important information. A mismatch is also seen in the area of informal neighbourhood help, which had become more relevant during the recent COVID-19 related lockdown. Younger people preferred services mediated through online apps, while many older people, especially in the age group 80 and over, did not use a smartphone or, for that matter, the internet. This moving of services into the digital realm without concern for older people’s needs and abilities creates new everyday difficulties for them.

Another possible barrier was the difficulty to access information on what support services were available for older people in a particular region. While there are many such resources, the availability of these services differs from region to region. Often, it is hard for older people and professionals alike to keep track of which services are available. While some communes operate contact points that provide such information to professionals and the general public, many do not. Also, a lack of coordination between the relevant services was reported, as illustrated by this quote:

From fall prevention to insurances to occupational therapy, everybody tends their own garden [. . .] we are rarely contacted by occupational therapists, ‘hey, do you have an idea’. There, it would be beneficial if you could integrate all these competencies in one centre. (Florian, accessibility consultant)

Participants advocated a more interprofessional approach to health and social care provision for older people to support autonomous living. One participant was familiar with a German equivalent to the idea of a ‘competence centre’ as described above:

We had this for example in [a neighbourhood in a large German city], we were a multiprofessional team there. We had somebody who knew about architecture and adaptations, we had a social worker who wrote all the grant applications for people, we had somebody for assistive devices – we even had an exhibit – and we had someone who knew about the care infrastructure, in- and outpatient options, etc. We were five people in one place and could provide help and support over the whole spectrum. (Eric, social worker)

In summary, participants noted the persistence of physical and other barriers that posed problems for older people with chronic conditions in their everyday lives, in terms of accessibility and usability. They also identified a lack of awareness among key persons, such as government officials, landlords and manufacturers, for these problems and the need to raise this awareness. Furthermore, they observed the emergence of new barriers, tied to societal digitization and the gradual move of services into the digital realm, while many older people – especially the one 80 years old and older – were not used to using smartphones and the internet. Lastly, finding the relevant services was not always easy for older people. A more collaborative approach among health and social care professionals could help improve older people’s access to relevant services.

### Theme 4: ‘What does “remaining living at home” really mean?’

A final theme that we identified in the data concerned the question what people really mean when they speak of ‘remaining living at home’. This point was addressed by two participants. Barbara granted that, in her experience, to remain living at home is the wish of most older people, but added also that it is the predominant discourse at the time. Barbara and Gloria raised the question what this meant to people exactly:

At the moment, everybody talks about how everybody wants to stay at home [. . .] But I always ask myself: what does ‘remaining living at home’ really mean? Is it about your own four walls, or about your social contact? Or is it about autonomy? Because that can differ a lot. (Barbara, architect)

‘What is it exactly, that makes a difference for you?’ (Gloria, occupational therapist)

The participants raise the question if the issue of ‘remaining living it home’ may be more complex, and require a broader, societal discussion that goes beyond the home – institution dichotomy, but rather asks the question what people actually need and how these needs are best met. Remaining in their current house or apartment may not always be the best solution for everybody; however, participants miss a more fundamental debate on this.

Some participants also deplored the fact that in their perception, there were few options other than staying at home or moving to a nursing home. Additionally, they thought there should be more other, intermediate options:

Maybe remaining at home is not always the best solution, maybe switching to another form of living is [sometimes] better. Because they are maybe not alone anymore and receive support, or have less inhibitions to accept other services, because they are easily available. And there, I am missing a fundamental debate. (Barbara, architect)

In summary, framing the question of how older people with chronic conditions want to and should be able to live as a choice between ‘at home’ as a positive outcome and ‘in an institution’ as a negative outcome may be an undue simplification of the matter. Forms of living that cater to older people’s needs and wishes, for example, to participate and to retain autonomy, may be more important. Also, they voiced that there is a need for intermediate forms of living – for instance apartments with auxiliary-assisted living services – that are still not common in Switzerland. Participants described the need for a shift in the public debate on the topic.

## Discussion

With this study, we aimed to examine how different stakeholders involved in health care, social care and housing for older people with chronic conditions view the difficulties this group faces in everyday life. Participants stated that physical and other barriers not only that challenge older people with chronic conditions showed to be very persistent, but also that many adaptations and support services exist to help them meet these challenges. However, older people’s access to services is shaped by their fear of losing autonomy, their socio-economic position, key stakeholders’ (e.g. landlords, government officials and product designers) lack of awareness of older people’s challenges, and a lack of information about and coordination between services.

The central importance that maintaining autonomy holds for older people has been extensively described in the literature (Ballmer and Gantschnig, 2023; Dendle et al., 2022; Eloranta et al., 2008; Ewart and Luck, 2013; Haak et al., 2007). How the fear of losing autonomy affects older people’s choices has not been examined this extensively. In a survey among 2155 Swiss people 65 years old and older, fear of losing autonomy and privacy concerns were the most important reasons for hesitating to use home care services (Dupraz et al., 2020), and it has also been reported that fear of losing autonomy is an important reason why older people do no report falls (Beaudette, 2011; Moylan and Binder, 2007). There is also limited evidence on the level of awareness of landlords, product designers and government officials regarding accessibility and usability of environments and products for older people, in spite of seminal documents like the WHO’s Global Age-friendly Cities Guide (WHO, 2007) and an extensive relevant user experience literature (e.g. Barnard et al., 2013; Czaja, 2019; Page, 2014).

That persons with a lower socioeconomic status face more difficulties accessing healthcare services has been described in the literature across a wide range of contexts (Caballo et al., 2021; McMaughan et al., 2020; Moscelli et al., 2018) and specifically for Switzerland (Boes et al., 2016; Spiess and Schnyder-Walser, 2018). Possible reasons are that people who are less educated have more trouble navigating the system, that people with less financial resources forego treatment for financial reasons, and that the healthcare system may not take into account the specific needs of people with a lower socio-economic status (Weber and Hösli, 2020).

The lack of knowledge about available services can be described as a function of health literacy, defined as ‘the degree to which individuals have the ability to find, understand and use information and services to inform health-related decisions and actions for themselves and others’ (Centre for Disease Control, 2022: 2). In a survey of 10,230 US residents aged 51 or older, people with low health literacy were ‘significantly more likely than individuals with adequate health literacy to delay or forego needed care or to report difficulty finding a provider’ (Levy and Janke, 2016: 936). Health literacy has been linked to socio-economic status. In a German survey of 475 adults aged 65 years and older, financial deprivation was the strongest predictor of limited health literacy. While this indicates that socio-economic status has an indirect effect on service use mediated by health literacy, the fact that certain services are not or only partially publicly funded (e.g. home adaptations) shows that there is also a direct connection between limited financial resources and access to services.

However, according to the participants in this study not only older people themselves but also professionals often lack the overview of available support services, and there is a lack of coordination among such services. Within the Swiss health care system, interprofessional collaboration in an outpatient setting is much harder to coordinate than in inpatient settings, partly due to a lack of established communication structures and remuneration opportunities (Meidert and Ballmer, 2020; Schmitz et al., 2017). The lack of coordination between services across different sectors, like health care and social care, is another challenge that has been reported for different national contexts (Melin Emilsson et al., 2020). In the Swiss context, this challenge is exacerbated by the fact that funding for health and social care is entirely separate. Other European countries where these services are both state funded, for example, the UK, are currently trending towards integrating health and social care even more (Department of Health and Social Care, 2022; Scottish Government, 2022).

Another issue that was raised by the participants, framed as ‘what does remaining living at home really mean’. Participants noted that the discourse about ageing-in-place was usually framed as a dichotomous choice between staying in one’s long-standing private home on the one hand and moving to an old people’s home on the other, with the former connoted as a ‘good’ outcome and the latter as a ‘bad’ outcome. The question was raised if this is not an undue simplification, and if a needs-based approach that addressed older people’s needs for autonomy and participation may not be more suitable, especially since there were intermediate forms of living between private housing and old people’s homes. In a recent report, the Swiss health observatory OBSAN (Werner et al., 2021) confirms that such intermediate structures are becoming more important in Switzerland, but that there are considerable regional differences.

Little is known about the degree to which the documented wish of many if not most older people to age in their familiar surroundings may be due to a fear of losing autonomy. In a study of 80 German and Swedish older people between 80 and 89 years of age who live in their private homes, the participants’ desire to ‘stay put’ was often articulated in direct contrast to the ‘final frontier’ (Löfqvist et al., 2013: 924) of moving to a nursing home. An exemplary quote reads, ‘I definitely want to stay alone as long as possible. I do not want to go into a nursing home’ (Löfqvist et al., 2013: 924). It is possible that the familiar home is sometimes merely a ‘signification of independence and autonomy’ – while, in reality, living in this home may not always be conducive to independence and autonomy, as discussed in *Theme 1* – in demarcation to moving to an institution as a *signification* of loss of autonomy. Moving the public discourse away from this supposed dichotomy and towards a more needs-based discourse may help older people reflect on what is the right place for them to age-in-place.

### Limitations

This study consists of only one single focus group, and therefore provides only a limited picture of relevant stakeholders’ understanding of the difficulties older people with chronic conditions living at home face in their everyday lives. While the analysis is based on a limited amount of data, we have used prolonged engagement with the transcript, regular peer debriefing and researcher triangulation to strengthen the trustworthiness of our analysis (Lincoln and Guba, 1985; Nowell et al., 2017). Also, the views of older people themselves are not present. The latter have however been described in our recent study that complements this one (Ballmer and Gantschnig, 2023).

### Implications for occupational therapy

Our findings are not only relevant for occupational therapists, who are deeply involved in providing home adaptations and assistive technologies to older people living at home, but also for other professionals who work directly with or are involved in shaping services and/or policies that affect older people (e.g. other health and social care professionals, politicians, community workers). Occupational therapists, as well as other professionals, can serve their clients better if they are aware of how fear of autonomy loss might affect their willingness to accept adaptations and services, as well as the need to advocate on behalf of their clients with key persons such as landlords, government officials and product designers. Lastly, recognizing how the socio-economical position of their clients shapes their choices, and how to navigate the system accordingly (especially in terms of funding options) may improve the quality of occupational therapy interventions.

## Conclusion

In this study, professionals involved in health care, social care and housing for older people with chronic conditions identified socio-economic position, fear of autonomy loss, a lack of awareness among key persons (e.g. landlords, government officials, product designers) and lack of information about and coordination between services as factors that shape the access older people have to adaptations and services that could support their autonomy and participation. Acknowledging the influence of socio-economic position may necessitate rethinking funding options for relevant adaptations and services. More research about how older people’s fear of autonomy loss and a lack of awareness of key persons shapes older people’s access to adaptations and services is needed in order to improve older people’s chances to autonomously live in the place of their choice for as long as possible.

Meanwhile, the lack of information about and coordination between services calls for innovations in community-based service provision in Switzerland. Finally, the participants’ statements suggest that moving public discourse about older people’s housing towards a discourse based more on what people actually want and need may help older people reflect on the right place for them to age-in-place.

**Key findings**

- Socio-economic status and fear of autonomy loss shape older people’s access to adaptations and services
- Key stakeholders’ lack of awareness of older people’s needs curtails the latter’s access to services

**What the study has added**

This study shows internal and external factors that can limit older people’s opportunities for autonomous living and documents calls for a more nuanced discussion of models of autonomous ageing.
